# Response of US psychiatric programs to the COVID-19 pandemic and the impact on trainees

**DOI:** 10.1186/s12909-022-03286-x

**Published:** 2022-04-01

**Authors:** Tyler Durns, Thomas Gethin-Jones, Eric Monson, Jennifer O’Donohoe

**Affiliations:** Department of Psychiatry, University of Utah, 501 Chipeta Way, Salt Lake City, UT 84108 USA

**Keywords:** COVID-19, Wellness, Burnout, Communication, Pandemic

## Abstract

**Background:**

Medical training program and hospital response to the COVID-19 pandemic has varied greatly and has impacted trainee well-being. Which factors have specifically related to trainee wellness, however, has not yet been examined in depth. The aim of the study was to understand trainee perspectives on the individual psychiatry trainee programs’ hospitals’ objective COVID-19 preparedness management. We also sought and to gauge how program changes, and general pandemic-related concerns, have been associated with trainee satisfaction and burnout.

**Methods:**

A cross-sectional survey study of psychiatric trainees was distributed electronically throughout the country via various psychiatry residency program listservs in April 2020. Statistical analyses were performed utilizing simple linear regression.

**Results:**

From 352 respondents (346 complete responses and 6 partial responses), the most frequent program changes were “decreased number of rotations requiring in-person patient care” and “increased call hours or duties.” Of pandemic-related concerns surveyed, the two greatest were “spreading COVID-19 to family/friends” and “co-residents’ burnout and anxiety.” A positive relationship was found between trainee satisfaction with perceived COVID-19 departmental response and comfort level of residents/fellows in expressing concerns with attending clinicians and department leadership.

**Conclusions:**

Since the start of the COVID-19 pandemic, trainees have experienced a variety of changes to trainee program policies and guidelines. Overall, poor communication and trainee dissatisfaction with departmental response correlated with concern of infection and anxiety/burnout. Insights garnered from this study could provide scaffolding for the best practices to reduce trainee physician anxiety/burnout for the current and future pandemics of this variety and magnitude.

**Supplementary Information:**

The online version contains supplementary material available at 10.1186/s12909-022-03286-x.

## Introduction

The global spread of SARS-CoV-2 (the virus causing COVID-19) has resulted in significant morbidity and mortality, requiring hospital systems around the world to quickly and frequently implement changes in preparation and management of disease proliferation [1]. Given decentralized best practice guidelines and mandates across governmental, nonprofit, business, and professional associations/organizations providing oversight, COVID-19 preparation and management has varied significantly [2–5]. Among healthcare providers in general, resident and fellow trainees are on the frontlines of the current pandemic and operating under various policies and guidelines depending on facility and need [6–8]. There is a dearth of compiled data from these trainees that could provide insight into best practices for this and potentially future pandemics of this variety and magnitude.

Despite rapidly evolving changes in response to the pandemic, little attention has been paid to how psychiatric practice, training programs, and trainees have been impacted. The delay in investigation is particularly impermissible considering that lower physician satisfaction has consistently been found to lead to poorer quality of care, as well as an increase in safety incidents [9]. In previous infectious disease pandemics, such as the Influenza A H1N1 of 2009 and HIV/AIDS pandemics, studies examining trainee comfort and safety were severely delayed for years. With regard to HIV/AIDS, it has been found that over one-third of residents were “very concerned” about contracting these illnesses from their patients, and those trainees with increased exposure correlated with training dissatisfaction [10]. In the H1N1 pandemic, trainees reported feeling obligated to work while ill themselves, leading to increased chance of transmission to coworkers and patients alike [11, 12]. Trainees are often viewed as the primary caretaker for patients and frequently have more individual contact than other treatment team members [6, 12], increasing both risk and individual concern. Furthermore, although trainees have been found to have had high adherence to hand hygiene practices and vaccinations, significant gaps in knowledge of proper utilization of (as well as availability of) personal protective equipment (PPE) for trainees has led to poor compliance and more potential for spread [11, 12]. Finally, studies agreed that adequate clinician-in-training preparation for how to properly protect themselves and others during a pandemic was lacking [11, 12].

In the early months of the pandemic, many medical residents and fellows responded with eagerness to be involved with care of COVID-19 positive patients, other trainees also expressed feelings of “fear, despair, and anxiety^”7.^ Concerns and fears varied, including contracting the illness, PPE shortages, the ability to adhere to rapidly changing guidelines, and feeling as though trainees are not being prioritized regarding receiving PPE^7^. Furthermore, the perceived pressure to avoid relinquishing duty and placing burden on colleagues has been exacerbated [9]. As summarized by one resident, “I have seen discontent, even anger, toward programs and our current healthcare system for our lack of preparedness. Perhaps most disheartening, there have even been a few who have expressed concern and questioned whether we, as residents, should be taking care of patients with COVID-19. As health care providers, I believe we have the right to be nervous, even fearful, about what is to come” [7]. As COVID-19 proliferates globally, so does trepidation within the next generation of clinicians.

The aim of the study, is to survey psychiatry and subspecialty trainees about their own training program’s initial response to the COVID-19 pandemic and to gauge how program changes, and general pandemic-related concerns, impacted trainee satisfaction and burnout.

## Methods

The survey was developed by the authors and was informed by existing United States Centers for Disease Control and Prevention (CDC) guidelines as well as recommendations on infection control precautions for healthcare workers, and suspected or confirmed COVID-19 positive patients. The survey, along with an informed consent agreement, were input into Qualtrics.xm software (Qualtrics, Provo UT) with the system logic set to prohibit progression of the study without consent. This survey was distributed by email individually to all 263 allopathic and osteopathic General Psychiatry Residency Program Coordinators and Directors individually, as well as to all programs via the American Association of Directors of Psychiatric Residency Training (AADPRT) Program Director listserv and Program Coordinator listservs, to distribute to resident trainees if deemed appropriate. Fellowship Directors were not directly contacted. The survey was open from April 17, 2020 to April 29, 2020 and anonymized data was retrieved on closure of the survey.

This study was found exempt by the University of Utah Institutional Review Board (IRB_00131976), with no formal ethics approval required due to the anonymous nature of the survey and its adherence to national guidelines, however, informed consent was still requested prior to completion of the survey. After consenting, respondents were then directed to a sixteen-question survey (see supplementary materials). The survey included socio-demographic questions relevant to training (region of program as defined by the AADPRT), year in training, number of trainees per class, inpatient sites requiring coverage, and the number of COVID-19 cases in the state in which they are practicing at the time the survey was completed. Study-specific questions regarding perceived departmental changes such as consult policy changes, call changes (including several approaches), policy implementations for suspected and confirmed COVID-19 patients, and policies involving PPE comprised the next section of the survey. Several trainee concerns were also examined via Likert scales, which, for the purposes of statistical comparison, were treated as an ordinal approximation of a continuous variable as all outcomes had at least five categories or were summed across multiple questions [13–16]. These scales measured trainee concern for contraction and spread of COVID-19 to patients and family members, perceived PPE shortage, trainee burnout, perceived overall risk to trainees compared to attending clinicians, trainee comfort in communicating concerns to leadership, and overall satisfaction of department response. Trainee respondents were also encouraged to communicate any further comments at the end of the survey.

Statistical analyses evaluated trainee concerns, including perceived program responsiveness, implemented changes, and comfort level in communicating with the leadership. Briefly, primary outcomes were converted to numeric scores across one or more Likert scales, including satisfaction of department response by trainees (5 levels, scored 0–4, with higher values indicating higher satisfaction), perceived risk of trainee burnout (two 4-level scales, scored 0–3, were additively combined to a final scale that is scored 0–6, with higher scores indicating greater perceived risk), and perceived trainee infection risk (five 4-level scales, scored 0–3, and 5-level scales, scored 0–4, were additively combined to a final scale that is scored 0–16, with higher scores indicating higher perceived risk). We examined the relationship between these scored outcomes and trainee region, number of residents per class, number of hospitals covered by trainees, perceived changes to consult requirements for trainees (yes/no), perceived changes to rotations or call expectations for trainees (yes/no), perceived overall change to trainee workload (converted to a numerical sum of changes with + 1 for each perceived increase in workload and -1 for each decrease in workload), perceived trainee changes in responsibility toward COVID negative and COVID positive patients (converted to a numerical sum of responsibilities for each patient group, + 1 for each additional responsibility), perceived changes in PPE policy by trainees (yes/no), and trainee comfort level communicating concerns to leadership (5-level scale, scored 0–4 with higher values indicating increased comfort).

All statistical analyses were performed using the R statistical framework [17] utilizing simple linear regression. Outputs were collected of Beta (β), standard error (SE), and *p*-value (*P*). All *p*-values were subjected to conservative Bonferroni correction for a total of 29 comparisons, with a significance threshold of 0.0017 (0.05/29 test) required for study-wide significance. All corrected *p*-values < 0.001 are presented as *corrected P* < *0.001* for clarity.

Any survey responses that were not answered by a given respondent or demonstrated clear numerical inaccuracy (such as PGY class sizes larger than any U.S. psychiatry programs) were declared as missing within the analyses. As numerical results regarding the current number of COVID-19 cases in each state were highly variable, even within the same AADPRT region, this question was excluded from the analyses. Results from the statistical evaluation of trainee responses were plotted as boxplots within R using the ggplot2 library [18]. Finally, free text comments provided as part of the survey were independently rated as subjectively positive, negative, mixed, or neutral between two raters (JO, TGJ). Only statements that were agreed upon as positive, negative, or mixed were included as examples in the discussion.

## Results

For the complete range of responses to the questionnaire and numerical scoring of responses, see supplementary Table S1. Table 1 summarizes demographic data obtained from the survey. A total of 352 responses were received, though 6 individuals did not have all questions answered. The number of trainees were then stratified by year, with the majority of respondents representing PGY years 1–4 (93.2%). Residents also reported the number of trainees per year (with 84.5% of reporting programs having 12 or fewer residents) and the number of hospital sites covered (with 87.6% having 3 or fewer sites of coverage).

**Table 1:** Demographic data of the survey respondents

	**Trainee Respondents (n)**	**Total Trainee Percentage (%)**
**Year**		
**PGY1**	74	21.08%
**PGY2**	91	25.93%
**PGY3**	83	23.65%
**PGY4**	79	22.51%
**PGY5**	16	4.56%
**PGY6**	2	0.57%
**Other**	6	1.71%
**N/A**	1	0.028%
**Total**	351	100.00%

Demographic information for the survey respondents. The ‘N/A’ category denotes missing responses to demographic questions in the survey

**Fig. 1:**
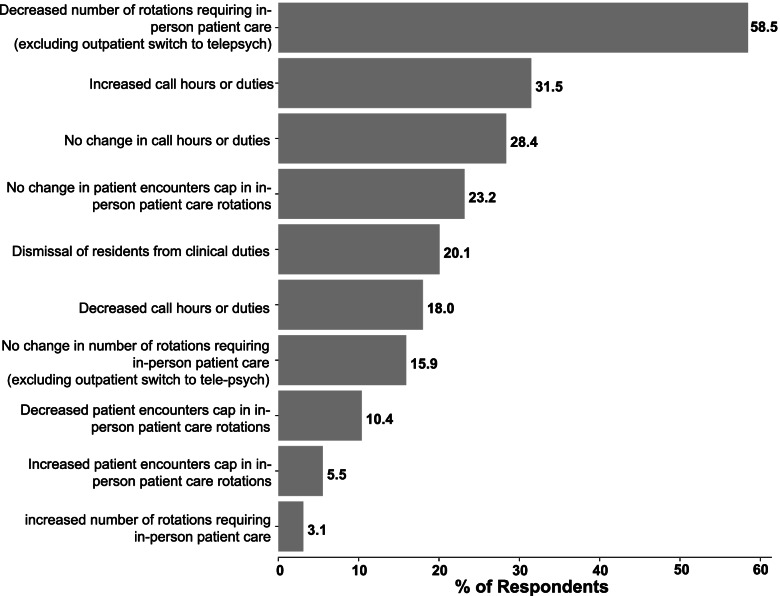
Program response/implementation. Breakdown bar chart showing relative frequency program responses/implementations

The remainder of the survey consisted of specific study data. 77.3% of respondents noted change in call or rotation structure as a result of the COVID-19 pandemic with more specific implemented changes being outlined in Fig. 1. Notably, 31.5% of residents reported increased call responsibilities or duties and 58.5% reported a decrease in their in-person patient interactions. 84.4% of trainees reported that their department had initiated policies against seeing non-urgent inpatient consults in-person. Regarding patients without a concern for COVID-19, 98.1% of trainees were expected to perform full psychiatric exams with 72.4% also providing physical examination of psychiatric patients, 33.1% were expected to do medical assessments and care for non-psychiatric patients, 33.4% were expected to perform rapid/code response, and 10.9% reported a new requirement of performing testing/swabbing. When treating patients with suspected or confirmed COVID-19, 93.1% of responding trainees reported being expected to perform psychiatric examinations and care, 36.3% reported an expectation to perform physical examination of psychiatric patients, 24.4% reported being expected to perform medical assessments and care for non-psychiatric patients, 30.2% expected to perform rapid/code response, and 13.0% of trainees reported being expected to perform testing/swabbing. Finally, trainees were asked whether there were policy changes to PPE use in seeing patients, with 83.0% of respondents reporting changes in policy due to current or anticipated shortages of PPE.

**Fig. 2:**
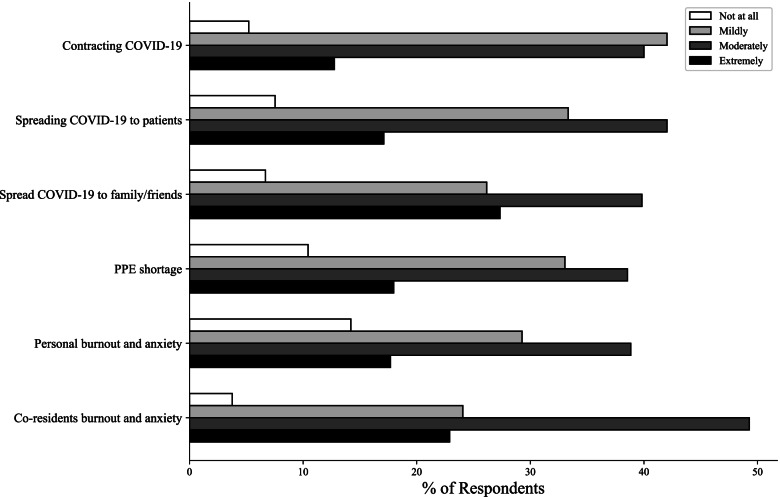
COVID-19 Related Concern. Bar graph demonstrating level of distress amongst COVID-19 related concerns

The next portion of the survey examined perceived trainee concerns regarding infection risk and burnout, with a summary of these results being shown in Fig. 2. Briefly, 94.5% of trainees felt they were at risk of contracting a COVID-19 infection, 83.5% felt there was a risk they would spread infection to other patients, 93.1% felt that there was a risk that they may infect family and friends, and 89.6% had concern about PPE shortages in their facilities. In addition, 85.8% of trainees had concern for personal burnout and 96.2% had concern for their co-resident’s risk for burnout. When asked to report the perceived risk of trainee infection compared to attending clinicians, 52.0% of responding trainees believed the trainee risk was higher. Respondents were asked to report their level of comfort communicating their concerns to attending clinicians or department leadership, with 77.3% of trainees feeling comfortable and 16.0% feeling uncomfortable. Finally, satisfaction with departmental response was evaluated, with 76.0% of trainees satisfied and 18.0% dissatisfied.

**Fig. 3:**
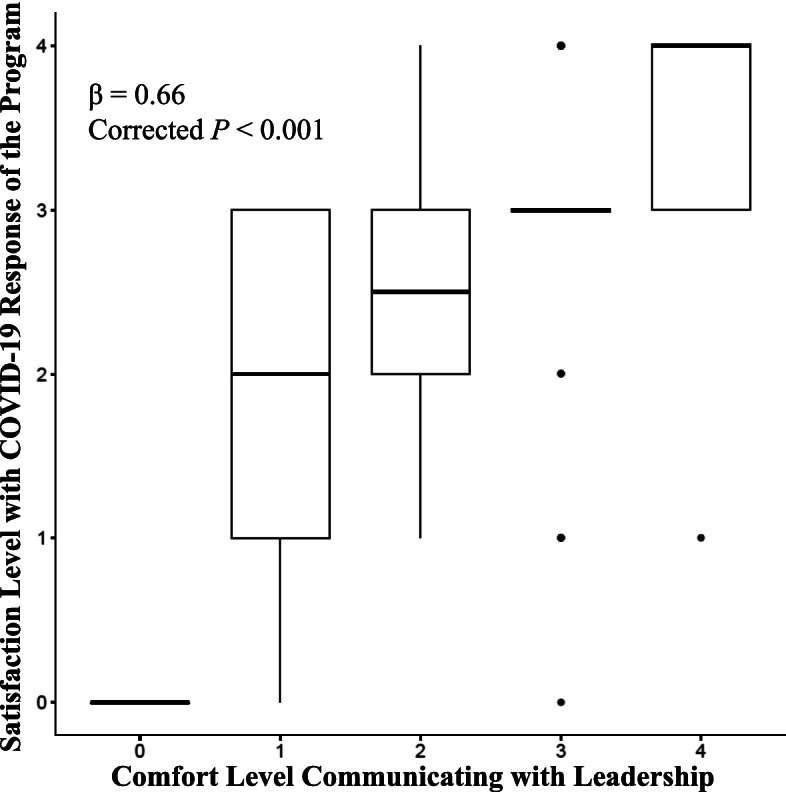
Box plot of Resident Rated Satisfaction with Program COVID-19 Response as Compared with Resident Rated Comfort Level in Communicating with Leadership. Box plot of the numerical values assigned to responses from survey questions 14 and 15 representing the horizontal and vertical axes, respectively. These questions evaluated the comfort level of resident/fellow in expressing concerns to, and communicating with leadership (Q14), and the level of satisfaction with current response (Q15), both rated from 0–4 with higher values representing more comfort and satisfaction, respectively

Twenty-nine statistical comparisons of the above response data were performed to identify important relationships in perceived trainee risks and trainee comfort level for communication of concerns to program leadership versus perceived program changes and trainee satisfaction regarding these changes (see supplementary Table S2 for complete results of these comparisons). One significant relationship in our study was the positive correlation of trainee comfort communicating with the program leadership and trainee satisfaction with program response (see Fig. 3; β = 0.66, SE = 0.039, corrected *P* < 0.001). Other significant results included comparisons of resident perceived risk of infection or burnout with satisfaction of department response (β = -0.14 and -0.24, SE = 0.019 and 0.037, and corrected *P* < 0.001 for both results, respectively) and comfort communicating with the department (β = -0.79 and -0.41, SE = 0.13 and 0.069, and corrected *P* < 0.001 for both results, respectively). Consistently, our study showed significant correlations of increased concern for both infection and burnout with decreasing satisfaction with both communication with leadership and program response.

### Trainee comments

Some of the subjectively positive comments (question 16) in the survey included themes of feeling well-supported, advocated for, safe, and informed. Trainees noted, “The communication from the Department has been outstanding. Every day we get updates. We were supplied with PPEs and had modules explaining how to use, reuse and take all possible safety precautions. Everyone is supportive of each other,” and “I feel that our department is doing the best that they can in a very tough situation. They have been wonderfully transparent.”

Negative concerns from the surveyed trainees included receiving “backlash,” or fear of being “fired,” for voicing their concerns. Some trainees reported feeling excluded from decisions regarding system changes that directly affected their work, or abandoned by their attendings, senior residents, or administrative authority figures. One trainee noted, “We have not been included in most conversations sbout [sic] clinical care and we have been excluded from many important clinical communications.” Others were concerned about the timeliness of response, stating “The slow response of my department has been extremely disheartening and made me question very heavily if I will be staying with this department upon residency completion.” In the most extreme examples, psychiatric care was no longer prioritized due to redeployment to other healthcare services, for example “I have been also deployed to Medicine Floors which have [sic] severely impacted my ability to do patient care with my outpatient psychiatry patients.”

Subjectively mixed responses to question 16 included themes of discordance between how different authorities were addressing their concerns. For example, “While I’m very satisfied with my program director’s individual response, our department leaders have hand [sic] difficulty taking unified, swift action.” Others noted positive changes over time, “I was initially not very satisfied with the response. However, we have a very vocal group of residents and are lucky to have a department willing to take our feedback.”

## Discussion

As the COVID-19 pandemic endures, so must clinicians. Given the established relationship with frequency of patient contact during pandemics with secondary post-traumatic stress and other psychological distress, trainees are particularly vulnerable [19–21]. The findings of this study indicate that training programs should assure healthy communication and transparent institutional changes to promote trainee satisfaction, decrease over-concern of infectious spread, and reduce anxiety/burnout. This is consistent with other studies on mental health outcomes for clinicians in previous and recent pandemics [22–25], as well as those emerging during the COVID-19 pandemic [26–28]. Differences in subjective experience as a result of pandemic-related mental illness in healthcare workers is an inherent and previously demonstrated barrier in examining healthcare worker perceptions of institutional response [29], which was also suggested by the general variation in trainee comments.

### Program response

Program responses varied across respondents. However, the most frequent perceived changes were decreased rotations requiring in-person patient care and extended call hours or duties. This could suggest efforts to reduce contact redundancy (such as both attending and trainee seeing the same patient, assuring the same trainee was seeing the same patients rather than multiple, etc.), a shift of resident specialty responsibilities during the pandemic, or contrasting approaches. It is unclear from this study whether program changes have impacted resident training, which must be weighed against trainee safety and public health interests. However, with the growing presence and proven efficacy of virtual-based psychiatric care [30–32], it appears likely that quality training may still be achieved when balancing these interests. The various combinations of changes may also be related to the number of cases in the program’s catchment area. Unfortunately, due to apparent inaccuracies in respondent COVID-19 case prevalence reporting, we were unable to test this. Given that our survey was directed only toward trainees and not directly to programs or leadership, our conclusions about the responses of specific US psychiatry training programs are limited to trainee perceptions rather than the full scope of implemented changes and should be interpreted considering this limitation.

### Trainee distress

Trainees expressed concern across all domains questioned. Notably the most extreme levels of concern (“extremely” and “moderately” concerned) were dominated by concern for others, with fear of co-resident burnout, spreading COVID-19 to loved ones, and transmission to patients being the most frequently reported. This again lends credence to previous claims on the importance of providing necessary materials (PPE) and proper education on how to best reduce transmission [11, 12]. Furthermore, it raises significant concern for trainee burnout and the implications this has on clinicians and patients alike, given consistent evidence that high levels of resident burnout lead to increased safety issues [9]. While respondents were less likely to report significant concern for themselves as compared to concern for others, this may be in part due to social desirability bias inherent to self-reporting. Finally, this raises significant concern for clinician welfare, supporting previous findings that hospital staff are reluctant to seek psychiatric/psychological care for themselves amid the COVID-19 pandemic [33], suggesting a continued need for attention to mental health of clinician trainees in the wake of the pandemic.

Among the relationships examined, trainee burnout negatively correlated with level of comfort communicating concerns to leadership and overall level of satisfaction with program response. Additional studies have noted similar findings in other specialty training programs. This suggests that similar principles may be applicable across medical training [34, 35]. Similarly, less concern for infectious spread (to self, loved ones, and patients) showed strong relationships with level of comfort communicating concerns to leadership and overall level of satisfaction with program response as well (Fig. 3). Together these highlight the importance of communication between leadership and trainees during a pandemic, as well as the importance of measures taken to mitigate concern. Satisfaction of department response was found to be inversely related to discomfort communicating concerns. Given these findings, it seems crucial for leadership to take a proactive approach to communication, both in collecting information from trainees and in the transparent dissemination of policies and plans.

The method of distribution in electronically distributing surveys to both program coordinators and directors for them to forward to their trainees was felt to be the best means to widely distribute the survey in a responsible way. However, to assure anonymity and further protect the respondent, we did not request trainees to report to which training program they belong. Thus, there is no way of knowing how many trainees received the survey, and no response rate can be calculated. Other limitations include inability of respondents to identify their region from the available choices, as they were not provided with AADPRT region definitions. While great efforts were made to promote anonymity and comfort, the inherent nature of a study requesting feedback on one’s employer entails a social desirability bias. Specific program/institution names were not requested to ensure the security of the respondents. Therefore, in addition to the known national limitations in testing and differences in testing policies, survey data collected cannot be accurately correlated to local disease prevalence [36, 37]. Some respondents also noted that they were not on psychiatry services (e.g., they were working on an internal medicine service) at the time of taking the survey which may have affected some of their responses. While some fellows were sent the survey by General Psychiatry Program Directors and Coordinators, fellowship listservs were not directly contacted. Therefore, the responses given by psychiatry fellows were limited. An additional limitation includes possible trainee misunderstanding of current institutional policies given the rapidity of policy changes and communication, and programs were not directly contacted to provide a more comprehensive understanding of these changes.

## Conclusion

Our study found a significant positive relationship between trainee comfort communicating with the program leadership and trainee satisfaction with program response. We also found a negative correlation between resident perceived risk of infection or burnout with satisfaction of department response and comfort communicating with the department. These findings highlight not only the importance of mitigating concerns of trainees through institutional modifications and good communication, but also the importance of an individualized approach with trainee concerns.

## Supplementary Information

**Additional file 1.**

## Data Availability

The datasets generated and/or analysed during the current study are not publicly available due to password encryption via Qualtrics.xm software but are available from the corresponding author on reasonable request.

## Abbreviations

PPE: Personal protective equipment
CDC: Centers for Disease Control and Prevention
AADPRT: American Association of Directors of Psychiatric Residency Training
Β: Beta
SE: Standard error
*P*: P-value
