# Supplementary material for: Response of US psychiatric programs to the COVID-19 pandemic and the impact on trainees
Source: BMC Med Educ. 2022 Apr 1;22:229. doi: 10.1186/s12909-022-03286-x (PMC8972686; doi:10.1186/s12909-022-03286-x)
Supplement: Supplementary file 1 — Additional file 1. [file 12909_2022_3286_MOESM1_ESM.docx]

**Supplementary Material**

***Survey***

I invite you to participate in a brief (less than 5 minutes) voluntary research study, “Emergency Response of US Psychiatric Programs in the Wake of the COVID-19 Pandemic.” If you have any questions, or if there is anything that is not clear, please do not hesitate to ask me any questions. Participation in this research is voluntary and **confidentiality will be maintained**. Participation in this study after review of this document serves as implied consent, thus no signature is required beyond selecting "I consent." Furthermore, participation is will have no impact on your grades, evaluations, standing in your programs, and your program faculty/staff will not know if you have or have not participated, nor your responses. Thank you so much for your time and helping share knowledge on how we as psychiatric providers across the nation can collectively better care for each other and our patients.

**1. What region best describes where your program is based from?**

- New England
- New York
- Mid-Atlantic
- Midwest
- Southeast
- California
- Far West

**2. Please select your PGY year**

- PGY1
- PGY2
- PGY3
- PGY4
- PGY5
- PGY6
- other

**3. How many residents are in your class?**

**4. How many inpatient psychiatry hospital sites are currently requiring coverage?**

- 1
- 2
- 3
- 4 or more

**5. Number of total COVID-19-positive cases in your state (view current numbers**[**here**](https://datastudio.google.com/embed/reporting/f56febd8-5c42-4191-bcea-87a3396f4508/page/GQFJB)**)**

**6. Has your department advised or initiated policies against seeing non-urgent inpatient consults in person?**

- yes
- no

**7. Have there been changes made to your call or rotation schedule as a result of the COVID-19 pandemic?**

- yes
- no

**8. If yes to question 7, which of the following changes have been made? Please select all that apply**

- increased call hours or duties
- no change in call hours or duties
- decreased call hours or duties
- decreased number of rotations requiring in-person patient care (excluding outpatient switch to telepsych)
- no change in number of rotations requiring in-person patient care (excluding outpatient switch to tele-psych)
- increased number of rotations requiring in-person patient care
- dismissal of residents from clinical duties
- increased patient encounters cap in in-person patient care rotations
- no change in patient encounters cap in in-person patient care rotations
- decreased patient encounters cap in in-person patient care rotations

× not applicable

**9. Under current policy, would you be expected to carry out any of the following clinical responsibilities in a patient WITHOUT concern for COVID-19? Please select all that apply.**

- physical examination and care for psychiatric patients
- psychiatric examination and care for psychiatric patients
- medical assessment and care of non-psychiatric patients
- rapid response/and or code response
- testing (swabbing) for COVID-19

**10. Under current policy, would you be expected to carry out any of the following clinical responsibilities in a patient WITH suspected or confirmed COVID-19 infection? Please select all that apply.**

- physical examination and care for psychiatric patients
- psychiatric examination and care for psychiatric patients
- medical assessment and care of non-psychiatric patients
- rapid response/and or code response
- testing (swabbing) for COVID-19

**11. Have there been changes in policy due to current or anticipated shortages of PPE (e.g., recommendations to reuse equipment such as N95 masks or face shields)?**

- yes
- no

**12. As of today, please rate your level of concern about the following issues**

|  |  |  | Not at all | Mildly | Moderately | Extremely |
| --- | --- | --- | --- | --- | --- | --- |
| Contracting COVID-19 |  |  |  |  |  |  |
| Spreading COVID-19 to patients |  |  |  |  |  |  |
| Spreading COVID-19 to family/friends |  |  |  |  |  |  |
| PPE shortage |  |  |  |  |  |  |
| Personal burnout and anxiety |  |  |  |  |  |  |
| Co-residents’ burnout and anxiety |  |  |  |  |  |  |

**13. How would you describe** **resident** **risk of contracting COVID-19 relative to your** **attendings**?

- much higher
- moderately higher
- same
- moderately lower
- much lower

**14. As of today, how comfortable do you feel communicating your concerns to attendings or department leadership?**

- extremely comfortable
- somewhat comfortable
- neither comfortable nor uncomfortable
- somewhat uncomfortable
- extremely uncomfortable

**15. As of today, how satisfied overall are you with your department's response to the COVID-19 pandemic?**

- extremely satisfied
- somewhat satisfied
- neither satisfied nor dissatisfied
- somewhat dissatisfied
- extremely dissatisfied

**16. Please include any additional comments or concerns:**

***Table S1­– Survey Responses***

| **Question** | | | **Responses** | | | | | | | | |
| --- | --- | --- | --- | --- | --- | --- | --- | --- | --- | --- | --- |
| Q1: What region best describes where your program is based from? | Mid-West (119) | California (28) | New York (39) | | Southeast (60) | | New England (22) | | Far West (48) | | Mid-Atlantic (34) |
| Q2: Please select your PGY year | PGY1 (74) | PGY2 (91) | PGY3 (83) | | PGY4 (79) | PGY5 (16) | | | PGY6 (2) | | Other (6) |
| Q3: How many residents are in your class? | | | Response range of 1-71, median of 8 | | | | | | | | |
| Q4: How many inpatient psychiatry hospital sites are currently requiring coverage? | | | 1 site (114) | 2 sites (110) | | | | 3 sites (80) | | 4 or more sites (43) | |
| Q5: Number of total COVID-19-positive cases in your state | | | Response range of 158-288000, median of 30812 | | | | | | | | |
|  | | |  | | | | | | | | |
| Q6: Has your department advised or initiated policies against seeing non-urgent inpatient consults in person? | | | Yes (292) | | | No (54) | | | | | |
| Q7: Have there been changes made to your call or rotation schedule as a result of the COVID-19 pandemic? | | | Yes (269) | | | No (79) | | | | | |
| Q8: If yes to question 7, which of the following changes have been made? | | | Responses converted numerically with +1 for each increase in work requirements and -1 for each reduction; response range of -4 to 3 with median of 0 | | | | | | | | |
| Q9: Under current policy, would you be expected to carry out any of the following clinical responsibilities in a patient WITHOUT concern for COVID-19? | | | Responses converted numerically with +1 for each required responsibility; response range of 1 to 5 with median of 2 | | | | | | | | |
| Q10: Under current policy, would you be expected to carry out any of the following clinical responsibilities in a patient WITH suspected or confirmed COVID-19 infection? | | | Responses converted numerically with +1 for each required responsibility; response range of 1 to 5 with median of 2 | | | | | | | | |
| Q11: Have there been changes in policy due to current or anticipated shortages of PPE (e.g., recommendations to reuse equipment such as N95 masks or face shields)? | | | Yes (287) | | | No (59) | | | | | |
| Q12.1-12.4 and Q13: Rate your level of concern about the following issues: Contracting COVID-19, spreading COVID-19 to patients/family/friends, and PPE shortage, and perceived risk vs. attendings | | | Responses rated with scores 0-3 (no concern to extremely concerned) or 0-4 (lower to higher risk) and summed with range 1-16 and median 9 | | | | | | | | |
| Q12.5-12.6: Respondents asked to "rate your level of concern about the following issues": perceived risk of personal and co-resident burnout | | | Responses rated with scores 0-3 (no concern to extremely concerned) and summed with range 0-6 and median 4 | | | | | | | | |
| Q14: As of today, how comfortable do you feel communicating your concerns to attendings or department leadership? | | | Responses rated with scores 0-4 (extremely uncomfortable to extremely comfortable) with range 0 to 4 and median 3 | | | | | | | | |
| Q15: As of today, how satisfied overall are you with your department's response to the COVID-19 pandemic? | | | Responses rated with scores 0-4 (extremely dissatisfied to extremely satisfied) with range 0 to 4 and median 3 | | | | | | | | |
| Q16: Please include any additional comments or concerns | | | No comment (299) | | | | Varied Response (53) | | | | |

***Table S2– Statistical Comparisons of Survey Responses***

| **Comparison** | **Beta** | **Std_Error** | **P_value** | **Corrected_P** |
| --- | --- | --- | --- | --- |
| Q15_Q14 | 0.66 | 0.039 | 1.09E-46 | 3.15E-45 |
| Q15_Inf_Risk | -0.14 | 0.019 | 6.31E-13 | 1.83E-11 |
| Q15_Burnout | -0.24 | 0.038 | 3.23E-10 | 9.37E-09 |
| Burnout_Q14 | -0.41 | 0.069 | 5.96E-09 | 1.73E-07 |
| Inf_Risk_Q14 | -0.79 | 0.133 | 6.49E-09 | 1.88E-07 |
| Q15_Q6 | 0.87 | 0.162 | 1.58E-07 | 4.58E-06 |
| Inf_Risk_Q6 | -1.48 | 0.436 | 7.72E-04 | 2.24E-02 |
| Q15_Q8 | -0.18 | 0.054 | 1.04E-03 | 3.03E-02 |
| Inf_Risk_Q8 | 0.40 | 0.141 | 4.46E-03 | 1.29E-01 |
| Burnout_Q8 | 0.19 | 0.075 | 1.01E-02 | 2.91E-01 |
| Q15_Q10 | -0.13 | 0.059 | 2.61E-02 | 7.56E-01 |
| Q15_Q3 | 0.01 | 0.011 | 5.65E-01 | 1.00E+00 |
| Q15_Q4 | 0.07 | 0.083 | 3.80E-01 | 1.00E+00 |
| Q15_Q7 | 0.22 | 0.145 | 1.27E-01 | 1.00E+00 |
| Q15_Q9 | -0.05 | 0.055 | 3.34E-01 | 1.00E+00 |
| Q15_Q11 | 0.20 | 0.163 | 2.10E-01 | 1.00E+00 |
| Burnout_Q3 | -0.02 | 0.016 | 1.86E-01 | 1.00E+00 |
| Burnout_Q4 | -0.07 | 0.114 | 5.33E-01 | 1.00E+00 |
| Burnout_Q6 | -0.42 | 0.227 | 6.60E-02 | 1.00E+00 |
| Burnout_Q7 | 0.31 | 0.198 | 1.18E-01 | 1.00E+00 |
| Burnout_Q9 | 0.01 | 0.074 | 9.43E-01 | 1.00E+00 |
| Burnout_Q10 | 0.15 | 0.078 | 5.94E-02 | 1.00E+00 |
| Burnout_Q11 | -0.16 | 0.220 | 4.73E-01 | 1.00E+00 |
| Inf_Risk_Q3 | -0.02 | 0.030 | 5.12E-01 | 1.00E+00 |
| Inf_Risk_Q4 | -0.02 | 0.218 | 9.38E-01 | 1.00E+00 |
| Inf_Risk_Q7 | -0.13 | 0.386 | 7.34E-01 | 1.00E+00 |
| Inf_Risk_Q9 | 0.12 | 0.145 | 3.95E-01 | 1.00E+00 |
| Inf_Risk_Q10 | 0.27 | 0.151 | 7.92E-02 | 1.00E+00 |
| Inf_Risk_Q11 | 0.14 | 0.427 | 7.44E-01 | 1.00E+00 |

**Key:**

Q3: Number of residents in respondent program

Q4: Number of inpatient psychiatry sites requiring coverage

Q6: Policies against seeing non-urgent consults

Q7: Call changes resultant COVID-19

Q9: Clinical responsibilities for a suspected/confirmed non-COVID positive patient

Q10: Clinical responsibilities for a suspected/confirmed COVID positive patient

Q11: Policy changes due to PPE shortages

Q14: Comfort communicating with leadership

Q15: Satisfaction with program response

Burnout: Perceived risk of trainee burnout

Inf_Risk: Perceived trainee infection risk
